# The synergistic inhibitory effect of combining therapies targeting EGFR and mitochondria in sarcomas

**DOI:** 10.18632/oncotarget.27416

**Published:** 2020-01-07

**Authors:** Xiaochun Wang, Reichelle X. Yeo, Philip J. Hogg, David Goldstein, Philip Crowe, Pierre J. Dilda, Jia-Lin Yang

**Affiliations:** ^1^Sarcoma and Nano-oncology Group, Adult Cancer Program, Lowy Cancer Research Centre, Prince of Wales Clinical School, Faculty of Medicine, University of New South Wales, Sydney, Australia; ^2^Department of Surgery, Prince of Wales Clinical School, Faculty of Medicine, University of New South Wales, Sydney, Australia; ^3^The Centenary Institute, NHMRC Clinical Trials Centre, Sydney Medical School, University of Sydney, Sydney, Australia; ^4^Department of Medical Oncology, Prince of Wales Clinical School, Faculty of Medicine, University of New South Wales, Sydney, Australia; ^5^Tumour Metabolism Group, Adult Cancer Program, Lowy Cancer Research Centre, Prince of Wales Clinical School, Faculty of Medicine, University of New South Wales, Sydney, Australia; ^*^These authors contributed equally to this work

**Keywords:** EGFR-targeted therapy, mitochondria inhibitor, tumour metabolism inhibition, sarcomas, combination therapy

## Abstract

Our group previously demonstrated that sarcoma cell lines were insensitive to epidermal growth factor receptor (EGFR) inhibitor gefitinib monotherapy. PENAO, an anti-tumour metabolic compound created in our laboratory, is currently in clinical trials. Considering the positive regulation of tumour energy production by both the EGFR signalling and tumour metabolism pathways, this study aimed to investigate the effect and mechanisms of combination therapy using gefitinib and PENAO in sarcoma cell lines *in vitro* and *in vivo*.

PENAO monotherapy reduced proliferation in 12 sarcoma cell lines. Combining gefitinib and PENAO resulted in synergistic inhibition in both a time- and dose-dependent manner in 3 sarcoma cell lines with less prominent monotherapy effects. Combined treatment significantly enhanced cell death and perturbed mitochondrial function. *In vivo* combination therapy with PENAO and gefitinib was non-toxic to mice and significantly delayed tumour growth and prolonged survival. At 20 days after treatment, tumours from the combination treated mice were significantly smaller than those from untreated and single drug treated mice. The survival curves also showed significant difference across and between groups.

The combination of PENAO and gefitinib *in vitro* and *in vivo*, shows promise as a treatment pathway in this poor outcome tumour.

## INTRODUCTION

Sarcomas are rare tumours of mesenchymal origin, capable of differentiating into different cell types such as connective tissue (fat, fibrous tissue, muscle), visceral tissue and bone [1]. Sarcomas account for less than 1% of adult solid malignancies [2], with 30% reported as osteosarcomas and 70% as soft tissue sarcomas (STS) [3]. Patients with a localized sarcoma have 83% chance for a five-year survival, whereas those sarcomas with lymph node involvement have a reduced prognosis of 54% and the worst prognosis is 16% for sarcomas spread to distant parts of the body [4]. The limitations in cure rates from excision of tumours and application of radiotherapy and conventional chemotherapeutic agents has increased the interest in treating sarcomas with targeted therapies [5].

**Figure 1:**
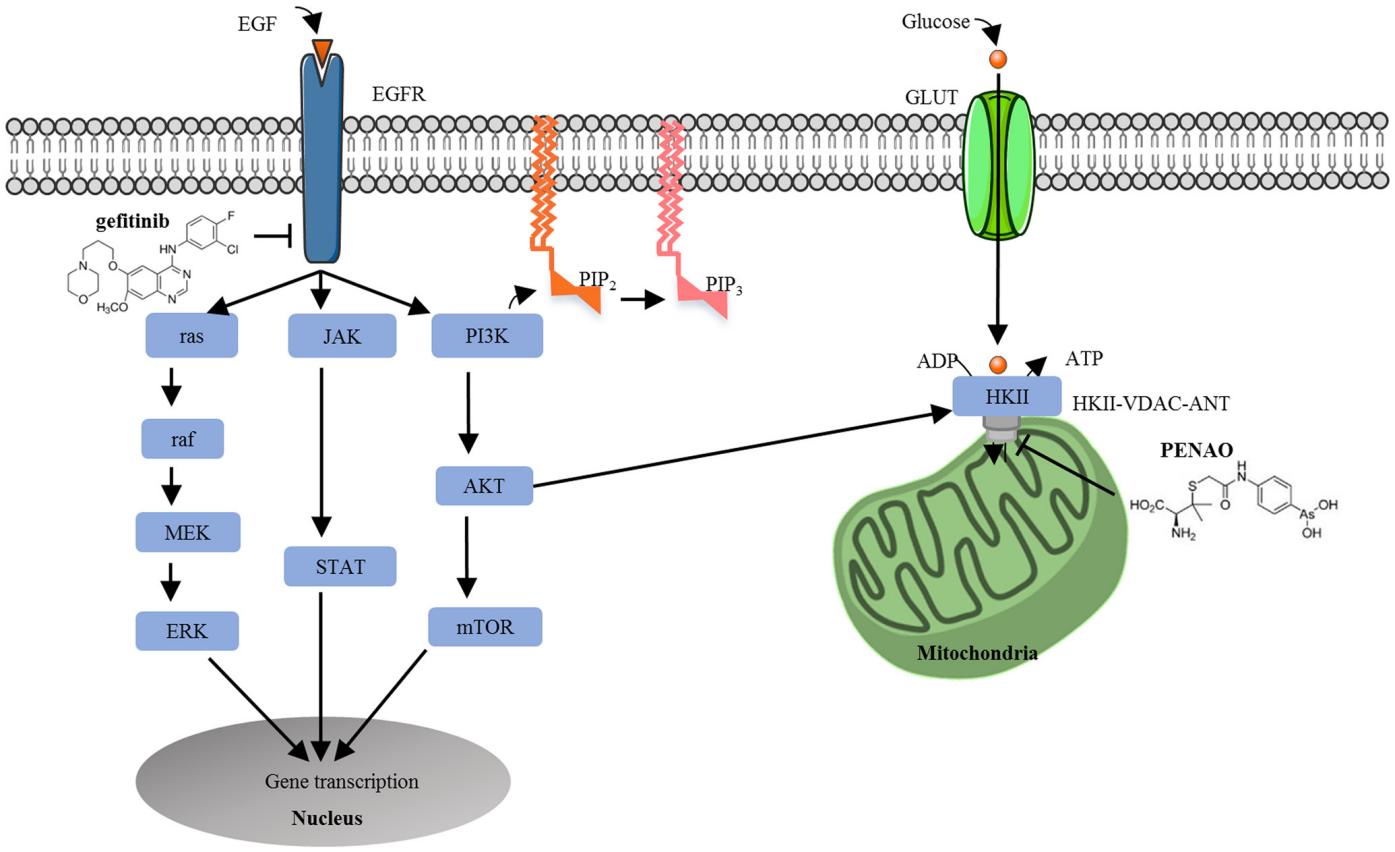
Cross talking between the epidermal growth factor receptor signalling and tumour metabolism pathways in tumour cells. In cancer cells, EGF or EGF-related ligands bind to EGFR and phosphorylate tyrosine kinase residues, activating three major signal transduction pathways: MAPK, PI3K/Akt and JAK/STAT, which promote cell survival. Upregulated PI3K/Akt activation also stimulates metabolic enzymes (i. e. hexokinase) to drive tumour metabolism. Cancer cells are reliant on mitochondrial metabolism for the increase of glucose to satisfy increased energy requirements. Glucose enters the cell via the glucose transporter (GLUT) to initiate glycolysis. HKII binds to voltage-dependent anion channel (VDAC) and adenine nucleotide translocase (ANT) to form a complex. Thus, the simultaneous inhibition of the EGFR signalling pathway and tumour metabolism pathway was explored. Gefitinib binds to the tyrosine kinase domain of EGFR to inhibit the activation of EGFR signalling whilst PENAO is transported across the plasma membrane and enters the mitochondrial matrix to interact with Cys^57^ and Cys^257^ residues in the ANT, perturb the mitochondrial permeability transition pore and decrease ATP delivery to the cancer cell. ****

Molecular-targeted therapies are highly specific towards proteins involved in tumour growth and progression [5]. The human epidermal growth factor receptor (EGFR/HER/erbB1) is a trans-membrane protein that belongs to a family of receptor tyrosine kinases (also including HER2/erbB2, HER3/erbB3 and HER4/erbB4) [6–9]. Ligand binding initiates several signalling cascades involving cell growth, proliferation, apoptosis and autophagy [10, 11] (Figure 1). The major signal transduction pathways are: ras/raf/mitogen activated protein kinase (MAPK), phosphoinositide 3-kinase (PI3K)/AKT/mammalian target of rapamycin (mTOR) and Janus kinase/signal transducer and activator of transcription (JAK/STAT) [12–15], all of which promote cell survival [16]. We and others have reported that EGFR was overexpressed in many types of osteosarcomas and STSs [9, 11, 17]. In conjunction with our recent published data (activated EGFR is an independent prognostic factor for overall and/or cancer specific survival) [18], these findings indicated that EGFR may be a promising anti-sarcoma target.

Gefitinib, an EGFR tyrosine kinase inhibitor, is approved as monotherapy for locally advanced or metastatic non-small cell lung cancer (NSCLC), a disease that overexpresses EGFR, after failure of treatment with standard chemotherapies [19] and has also shown efficacy in combination with chemotherapy [20]. Unfortunately, there are limitations due to both primary and acquired resistance [21–23]. By contrast, monotherapy with gefitinib did not significantly prevent cell proliferation in a preclinical synovial sarcoma model [24] and showed low response rates and short disease control in a phase II clinical trial [25]. Our group has reported that seven STS cell lines were insensitive to gefitinib monotherapy despite blockade of phosphorylated EGFR (pEGFR) and downstream signal transducers [26]. However, we demonstrated that the addition of a STAT3 inhibitor achieved synergistic anti-tumour effect in cell culture as well as in a fibrosarcoma xenograft mouse model [26], supporting investigation of additional combinations that may benefit sarcoma patients.

An extensively studied downstream pathway of EGFR is the PI3K/AKT/ mTOR pathway, critically involved in the regulation of cell apoptosis [27], autophagy [10], proliferation [12, 28] and metabolism [29]. Dysregulation of this pathway plays a major role in many different cancers [30, 31]. Constitutively activated PI3K/AKT signalling can directly induce translocation of glucose transporter GLUT to the plasma membrane, as well as stimulate metabolic enzymes such as hexokinase (the first enzyme in the glycolytic pathway) [32]. Cancer cells exhibit an aberrant metabolic phenotype known as the Warburg effect, where ATP generation via oxidative phosphorylation shifts to glycolysis, irrespective of oxygen availability, thus demanding a higher rate of glucose uptake [33, 34]. Therefore, EGFR/PI3K/AKT signalling enhances glucose entry and glycolysis to drive tumour metabolism (Figure 1).

Mitochondria play an important role in regulating energy metabolism, superoxide production and apoptosis [35]. Because they lie downstream in target pathways, they are less prone to acquiring mutations and tumour resistance [36–38]. Within the mitochondria, a promising anti-cancer target is the HKII (type II hexokinase)-VDAC (voltage dependent anion channel)-ANT (adenine nucleotide translocase) complex that spans the outer- and inner-mitochondrial membrane [39]. This transporter links the processes of glycolysis, oxidative phosphorylation and mitochondrial-mediated apoptosis in cancer cells [39, 40]. MPTP (mitochondrial permeability transition pore) centres on ANT that interacts with outer membrane VDAC [37]. In cancer cells, upon AKT activation, HKII translocates to the outer mitochondrial membrane and binds to VDAC, which interacts with ANT of the inner-mitochondrial membrane, and so preferentially accesses ATP produced by mitochondrial oxidative phosphorylation and phosphorylates glucose to produce glucose 6-phosphate, mediating the first step of glycolysis. The HKII-VDAC-ANT complex also plays important roles in acquiring resistance to apoptosis via blocking the binding of the pro-apoptotic molecule Bax to VDAC [41, 42]. Therefore, the inactivation of HKII-VDAC-ANT is an appealing anti-metabolic target, as blocking it inhibits HKII’s preferential access to newly synthesised ATP, increases superoxide production, triggers mitochondrial depolarisation and initiates apoptosis [39, 40, 43–45].

The discovery of significant efficacy of arsenic trioxide for the treatment of acute promyelocytic leukaemia [46, 47] encouraged investigations of inorganic and organic arsenical-based drugs (AsBD) for the treatment of other types of cancers. Evidence indicated that arsenic trioxide oxidizes ANT matrix cysteines, mediating oxidative stress, increased superoxide formation, MPTP opening and consequently cell death [40]. There are multiple ongoing clinical trials utilising AsBD as single agent or in combination therapy (with other chemotherapies) for the treatment of various haematological and solid tumours [48].

4-(N-(S-penicillaminylacetyl) amino) phenylarsonous acid (PENAO), a second generation synthetic trivalent organic-arsenical compound created in our laboratory, intrinsically blocks cell proliferation *in vitro* and tumour growth *in vivo* by perturbing mitochondrial function [45, 49–51]. PENAO’s trivalent arsenical moiety cross-links unpaired cysteine residues Cys^57^ and Cys^257^ located on the peptide loop of ANT that protrude into the matrix [39]. This leads to metabolic perturbation, swelling of the inner mitochondrial membrane, rupture of the outer membrane and cell death [38, 40, 52]. PENAO completed a phase I trial in patients with solid tumours refractory to standard treatment (trial ID: ANCTRN12612000908831). PENAO can be safely administered in humans and will be further explored in a phase IB trial to determine a dosing schedule [53].

Considering the crosstalk between EGFR signalling and metabolism pathways (Figure 1), in this study we explored the EGFR-targeted therapy combined with our mitochondrial inhibitor PENAO. We hypothesized that the simultaneous use of EGFR-targeted therapy with the tumour metabolism inhibitor PENAO would intrinsically double-block tumour metabolism and energy supply by targeting glycolysis via EGFR-PI3K/AKT-HKII signalling [54] whilst perturbing mitochondrial oxidative phosphorylation via PENAO-ANT, as well as induce the mitochondria-mediated apoptosis in cancer cells [38, 45]. There are a few published articles on synergism of arsenic-based drugs with EGFR inhibitors in cancer [55–58], but none in sarcoma models. The present study aimed to [1] investigate the effect of PENAO and gefitinib combination therapy on cell proliferation in a panel of 12 sarcoma cell lines [2], identify the mechanisms of cell death [3], assess tumour metabolism activity of sarcoma cell lines, and [4] investigate *in vivo* therapeutic effect of this combination treatment in Balb/c/nude mice bearing orthotopic human fibrosarcoma xenografts.

## RESULTS

### Anti-proliferative activity of PENAO and gefitinib monotherapy on 12 sarcoma cell lines

Prior to running end-point proliferation assays, sarcoma cell proliferation was recorded in real-time with the xCELLigence RTCA MP Analyzer (Roche, Mannheim, Germany). These preliminary experiments (data not shown) allowed us to determine the optimal time when end-point proliferation assays remain in exponential growth conditions for each cell line.

Anti-proliferative activity of PENAO and gefitinib as single agent treatment on sarcoma cell lines was determined by MTT endpoint assay after 72 hours of treatment (Table 1). IC_50_ values were calculated as concentration of drugs responsible for inhibition of proliferation by 50% [59] and results are presented as a mean ± standard deviation of at least 3 separate experiments performed with duplicate samples for each treatment. IC_50_ values for PENAO ranged from 1.1 µM to 7.3 µM, with HT1080 and SW872 being the most and least sensitive cell lines towards the drug. Consistent with our previous crystal violet assay in STS [26], IC_50_s of gefitinib determined by MTT assay in both STS and OS were between 14.0–30.0 µM, indicating insensitivity to gefitinib monotherapy, unlike a previously described *in vitro* lung cancer study (sensitivity threshold of gefitinib: IC_50_ ≤10 µM) [60].

**Table 1:** IC_50_ values of Gefitinib and PENAO for proliferation arrest of soft tissue sarcoma and osteosarcoma cell lines

Type	Cell line	Gefitinib IC_50_ (µM)	PENAO IC_50_ (µM)
STS	449B	28.4 ± 2.1	5.4 ± 1.4
	778	23.4 ± 3.0	3.8 ± 2.2
	GCT	14.0 ± 0.1	4.0 ± 0.7
	HT1080	15.3 ± 4.7	1.1 ± 0.3
	SW872	22.6 ± 6.5	7.3 ± 0.4
	SW982	15.4 ± 2.2	3.2 ± 0.4
Osteosarcoma	143B	21.5 ± 1.4	5.2 ± 1.9
	HOS	15.2 ± 1.5	5.0 ± 1.2
	MG63	22.9 ± 0.8	4.8 ± 0.7
	Saos-2	17.7 ± 0.3	5.3 ± 0.4
	SJSA	30.0 ± 4.4	4.7 ± 1.0
	U2-OS	19.3 ± 0.1	2.8 ± 1.2

### Effect of PENAO combined with gefitinib on the proliferation of 3 selected sarcoma cell lines

One osteosarcoma (HOS) and two soft tissue sarcoma (HT1080 and SW982) cell lines were chosen for combination study. HOS was chosen as it is the most sensitive osteosarcoma to gefitinib, whilst STSs HT1080 (fibrosarcoma) and SW982 (synovial sarcoma) were chosen as they were the most sensitive to PENAO. Combination treatments on proliferation were carried out in real-time (xCELLigence MP) and end-point (MTT) assays.

### Real-time response of sarcoma cells to single or combination drug therapy

**Figure 2:**
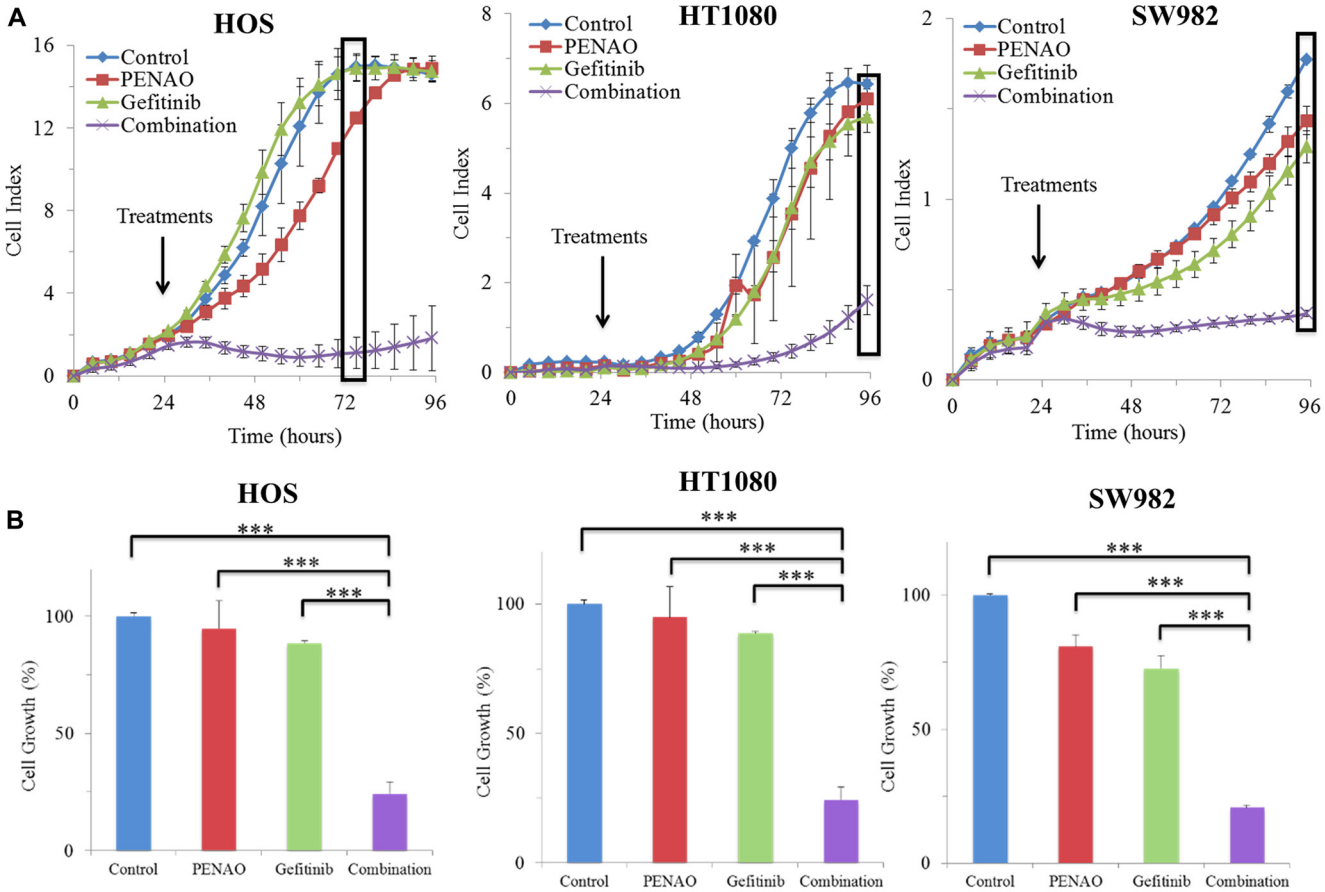
Real-time report of combination therapies significantly induced anti-proliferation in 3 sarcoma cell lines. xCELLigence real-time cell proliferation analysis of HOS, HT1080 and SW982 cell lines was recorded in real-time (**A**). 24 hours after seeding, cells were treated with PENAO, gefitinib and combination. Cell index is automatically measured in electrical impedance to represent cell status. Results are presented as a mean ± standard deviation. Results are representative of 2 experiments performed in triplicate samples for each treatment. (**B**) Combination treatment is compared to control and single treatments at an optimal time of the exponential cell growth phase on HOS at 48 hours after treatment (72 hours after seeding), and on HT1080 and SW982 at 72 hours after treatment (96 hours after seeding). ^***^ *p* < 0.001.

Combined treatments were performed at fixed ratios (1:1 in terms of IC_50_ doses). The real-time response of these three cell lines indicated that the concurrent combination treatments displayed significantly more potent inhibition of proliferation than control and single agents (*p* < 0.001), at about 48-hour post-treatment on HOS and at near 72-hour on HT1080 and SW982 (Figure 2).

### End-point dose response of sarcoma cells to single or combination drug therapy

**Figure 3:**
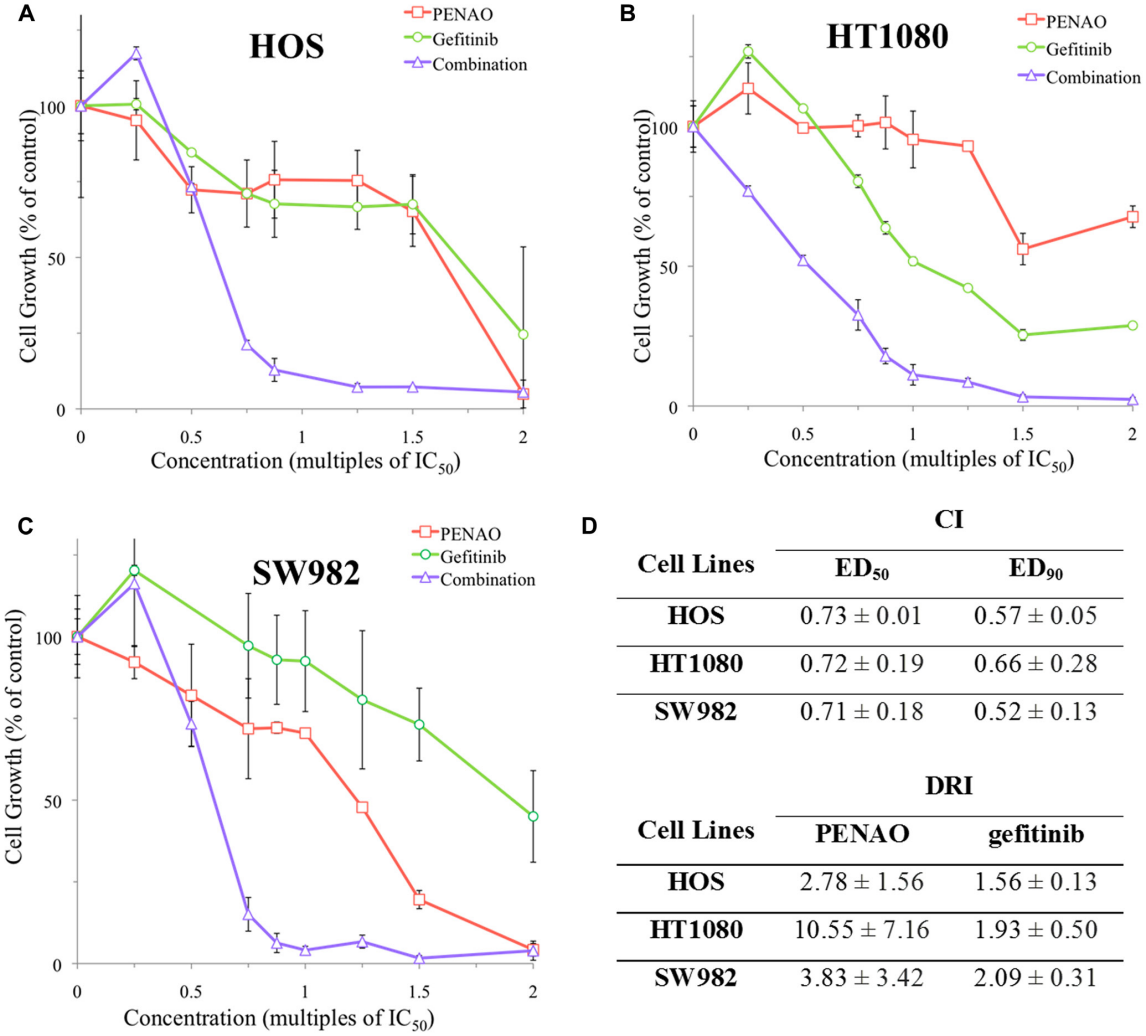
Endpoint analysis of combination treatments synergistically induced anti-proliferation in 3 sarcoma cell lines. A representative MTT endpoint graph is displayed for HOS (**A**) after 48 hours as well as HT1080 (**B**) and SW982 (**C**) after 72 hours of contact with PENAO, gefitinib and fixed ratios of combination treatment. (**D**) Combination index (CI) and dose reduction index (DRI) values were determined for 3 sarcoma cell lines using CalcuSyn software. Effective dose (ED_X_) is the amount of drug required to produce an effect on X% of the cell population. Dose reduction index (DRI) is the measure of fold each treatment in a synergistic combination can be reduced. Results are presented as a mean ± standard deviation. Graphs displayed are representative of at least 3 experiments performed in triplicate samples for each treatment.

To confirm the observations made in real-time proliferation assay and to determine the parameters of combination treatment on sarcoma cell proliferation, concurrent combinations were also performed using the MTT end-point proliferation assay on HOS, HT1080 and SW982 cell lines (Figure 3A, 3B and 3C). Calculation by CalcuSyn software (Biosoft, Cambridge, UK) showed that combination treatments were synergistic (combination index CI < 1) in terms of proliferation inhibition on HOS, HT1080 and SW982 (Figure 3D). In addition, dose reduction index (DRI) levels were calculated for these 3 cell lines and demonstrated that the combination therapy reached 1.56 to 10.55-fold dose reduction.

### Cell death induction by single or combination drug therapy

**Figure 4:**
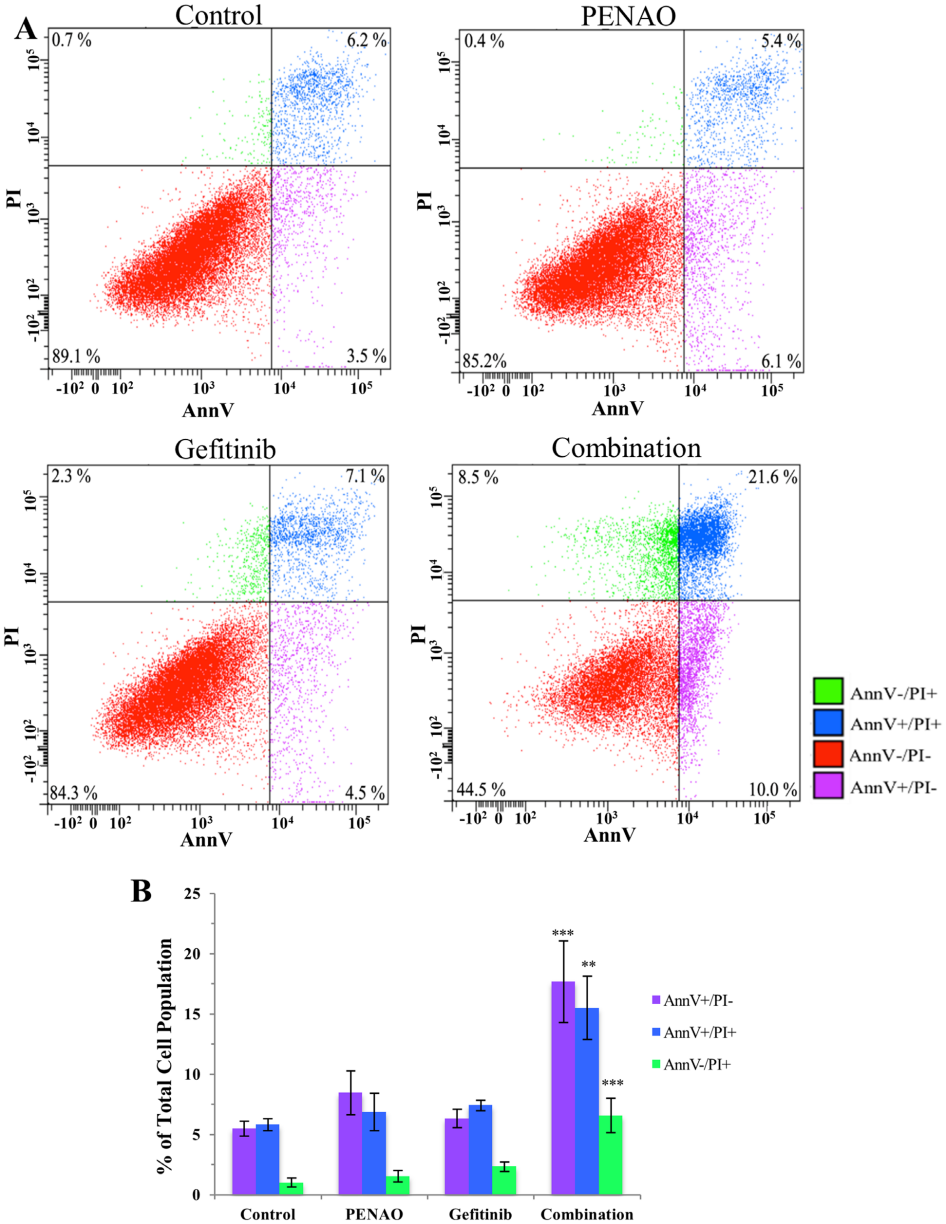
Combination therapy significantly induces cell death via apoptosis and necrosis. HOS cells were treated with vehicle control, PENAO and gefitinib as single drugs and in combination, then stained for early apopotosis (AnnV+/PI-), late (AnnV+/PI+) apoptosis and necrosis (AnnV-/PI+). (**A**) AnnV/PI density plot of one representative experiment comparing the treatment groups on HOS cells after 24 hours. (**B**) Early apoptotic, late apoptotic and necrotic cell populations in the combination treatment group were compared to the PENAO and gefitinib monotherapy groups and control group. Results are presented as a mean ± SEM of 3 experiments performed with duplicate samples. ^**^ *p* < 0.01; ^***^ *p* < 0.001.

For cell death analysis, concentrations of single agents were chosen to have limited effects. In these conditions, the impact of the combined treatment was easily demonstrated. HOS cells were chosen for flow cytometry based on the quality of combination treatment measured in proliferation assays. Treated HOS was analysed with AnnV/PI staining for early apoptosis (AnnV+/PI-), late apoptosis (AnnV+/PI+) and necrosis (AnnV-/PI+) (Figure 4A for one representative experiment). Cell populations in the PENAO and gefitinib monotherapy groups and combination treatment group were compared to the control. The data from triplicate experiments (Figure 4B) demonstrated that no single treatments (PENAO 8.5 ± 4.5%, gefitinib 6.3 ± 1.8%; *p* > 0.05) induced a significant increase in the early apoptosis cell population when compared to control. However, a significant increase of this population was measured in the combination group (17.7 ± 2.9%, *p* < 0.001). In terms of late apoptotic cells, single treatments had no significant effect compared to control (PENAO 6.9 ± 3.8%, gefitinib 7.4 ± 1.1%, *p* > 0.05), whereas the combination group 15.5 ± 6.4% (*p* < 0.01) was significantly higher than control. Similarly, cell necrosis was not induced by single treatments (PENAO 1.53 ± 1.1%, gefitinib 2.3 ± 1.0%, *p* > 0.05), but combination treatment was responsible for a modest but significant increase of the necrotic cell population (6.6 ± 0.5%, *p* < 0.001).

### Perturbation of cellular metabolism induced by single or combination drug therapy

**Figure 5:**
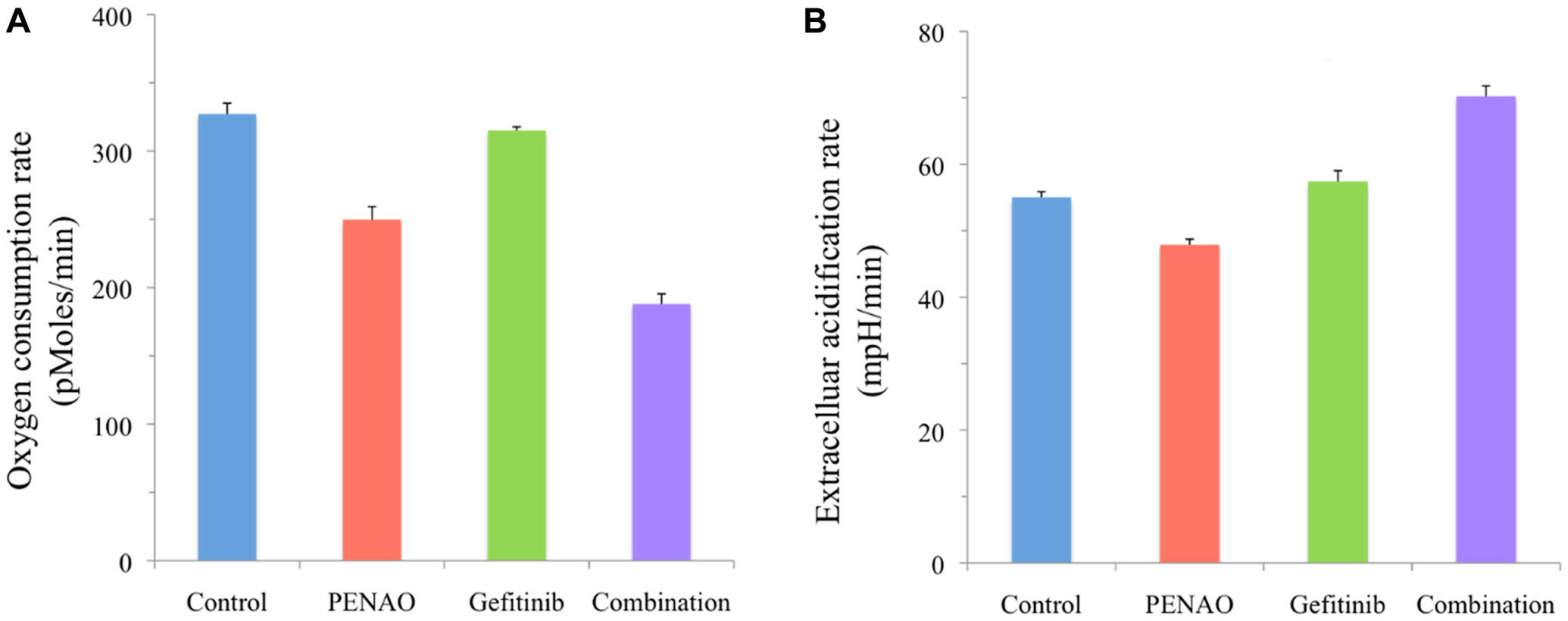
Combination treatment significantly affects HOS cell metabolism. HOS osteosarcoma cells were treated with vehicle control, PENAO and gefitinib (1.5 × IC_50_ concentrations) as single drugs and in combination. Metabolism was analysed using Seahorse XF^e^24 plate Analyzer (BioScience, Hohenkammer, Germany) after 24 hours of treatment. Measurements of HOS osteosarcoma cell (**A**) oxygen consumption rate and (**B**) extracellular acidification rate were recorded and normalised using the viable cell count. Results are representative of 2 distinct experiments performed at least in duplicate (control and combination in triplicate) samples for each experiment.

To investigate the effect of combined treatment on sarcoma cell metabolism, mitochondrial function was tested. HOS osteosarcoma cells were chosen for this experiment based on the promising combination profile exhibited in proliferation assays. Concentrations of single agents were chosen to have limited effect on metabolism. HOS cells were treated for 24 hours followed by the measurement of oxygen consumption rate (OCR) (Figure 5A) and extracellular acidification rate (ECAR) (Figure 5B). Combination treatment decreased more OCR compared to the control, PENAO and gefitinib single treatment groups, and as a consequence, ECAR increased more in the combination group compared to other treatment groups.

### Significant anti-tumour growth induced by concurrent treatment with gefitinib and PENAO in an orthotopic human fibrosarcoma xenograft nude mouse model

**Figure 6:**
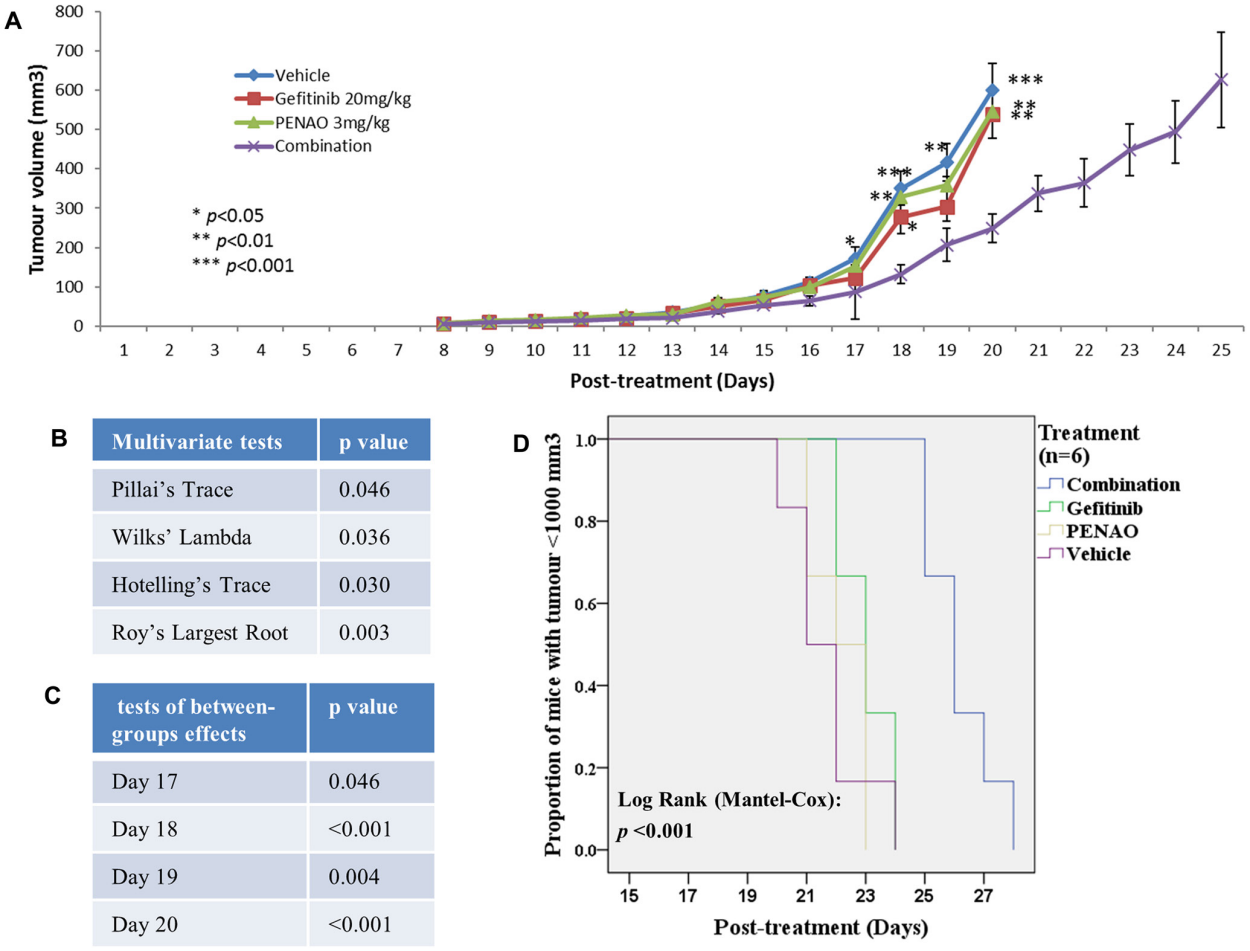
Combination therapy significantly enhanced the fibrosarcoma growth inhibition and delay in mouse model. 0.1 × 10^6^ HT1080 cells were injected intramuscularly into the right posterior thigh musculature of each nude mouse. On 24 hours post inoculation of tumour cells, all mice started treatment with vehicle, 20 mg/kg gefitinib (gavage), 3 mg/kg PENAO (pump) or combination once daily (*N* = 9–10 per group). (**A**) Tumour growth curve indicated that the combination therapy synergistically enhanced the fibrosarcoma growth inhibition. ^*^ *p* < 0.05 vs combination, ^**^ *p* < 0.01, ^***^ *p* < 0.001. The statistical analysis was done using IMB SPSS statistics 24 for the multivariate tests (**B**) and tests of between-subjects effects (**C**). (**D**) Kaplan-Meier survival curve of human fibrosarcoma xenografted mice comparing single or combination therapy (*n* = 6 per group). The difference was significant (log-rank test, *p* < 0.001).

To extend the investigation to *in vivo*, we used a human fibrosarcoma HT1080 xenograft nude mouse model, which has been successfully established in our group. After 24 hours intramuscular inoculation of HT1080, all mice were randomly divided into 4 groups (*n* = 9 or 10 per group) and treated with vehicle control, 20 mg/kg gefitinib (gavage, daily), 3 mg/kg/day PENAO (micro-osmotic pump) or combination with gefitinib plus PENAO. The dose of gefitinib was specifically selected so that its independent effect on tumour growth inhibition would be modest and the concentrations was clinically achievable [26]. Clinically relevant and achievable doses of PENAO ranges are yet to be revealed as clinical trials are still undergoing. On day 10–11 after treatment, tumours of about 3–4 mm in diameter were formed in all groups. Throughout the whole experiment, there was no anti-sarcoma effect using 3 mg/kg PENAO or 20 mg/kg gefitinib alone (*p* > 0.05). From day 17, the combination therapy significantly delayed the tumour growth, compare to vehicle control and monotherapy groups (Figure 6A, 6B and 6C). The tumour growth delay observed over two tumour volume doubling time was 3 days when the combination therapy was employed. On the 20th day post-treatment, tumours (248 mm^3^) from the combination treated mice were significantly smaller (tumour growth inhibition TGI = 59%) than those from untreated (599 mm^3^) and single drug treated (545 and 538 mm^3^) mice (*p* < 0.001, *p* = 0.005 and *p* = 0.004, respectively for combination versus vehicle, gefitinib and PENAO).

To show the beneficial effect of combination therapy, each individual mouse was sacrificed once its tumour reached about 1000 mm^3^. The Kaplan-Meier curve (Figure 6D) shows that combination therapy had significantly prolonged tumour growth delay (mean of time reaching volume of 1000 mm^3^ [days]: vehicle: 21, gefitinib: 23, PENAO: 22 and combination: 26; *p* < 0.001).

**Figure 7:**
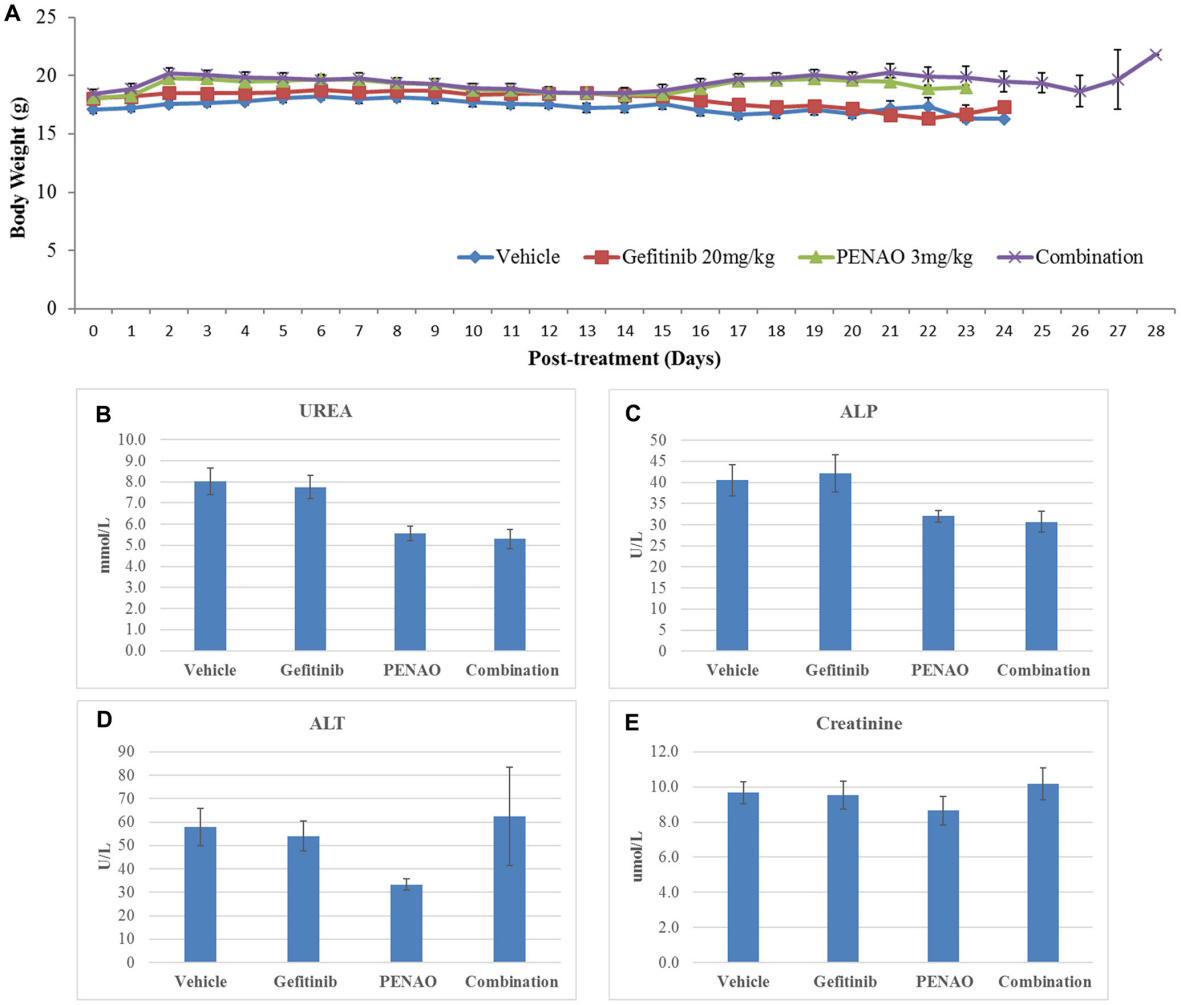
Gefitinib and PENAO alone and in combination were safe and well-tolerated by nude mice. (**A**). Mouse body weights were measured daily and no difference between untreated and treated groups were found. Quantitative data show means ± standard error of the mean (SEM). All *p* values (compared to vehicle control) >0.05. (**B**–**E**) Toxicity analysis of serum biomarkers after administration of gefitinib and PENAO alone and in combination.

Importantly, all treated mice tolerated PENAO and gefitinib well, showing no obvious signs or symptoms of distress or toxicity. All groups showed no body weight loss more than 20% and there was no significant difference in body weight changes between vehicle control and treated groups (Figure 7A, *p* > 0.05). All tumours and organs (lung, heart, liver, spleen and kidney) were harvested at the end points and showed no macromorphological and histological abnormalities (data are not shown). To further confirm whether toxicity was induced followed by the treatment, the blood was collected after sacrificing the mice to examine liver and kidney function. All four serum biomarkers (UREA, ALP, ALT and Creatinine) did not show significant increase after mono- or combination therapy (Figure 7B, 7C, 7D and 7E, *p* > 0.05,), supporting that the use of PENAO and gefitinib alone or in combination was safe and well-tolerated by nude mice.

## DISCUSSION

This study is the first of its kind to evaluate the effect and mechanisms of combining gefitinib and PENAO in the treatment of sarcomas. Results show combination therapy synergistically decreased cell proliferation in both time- and dose-dependent settings, significantly enhanced apoptosis, and perturbed mitochondrial function of sarcoma cells. This combination therapy has also demonstrated strong tumour growth inhibition without host toxicity in an *in vivo* experiment in nude mice, supporting future studies to validate this new combination therapeutic approach as a pathway to clinical trials. We chose a broad cell line panel to reflect the diversity of cell type and biology in sarcomas, using both osteosarcoma and several types of STS. These cell lines reflect common subtypes seen in clinical practice. Given our focus on identifying an approach with potentially broad activity in multiple sarcoma subtypes, we focused upon cell lines in which we have published data [61] showing EGFR overexpression, supported by the clinical results from two cohorts of tumour samples from patients with soft tissue sarcomas [9, 18] that found EGFR was overexpressed by 78% of the samples across different subtypes of STS. This suggests that EGFR is a therapeutic target for treatment of STS; which is independent of histologic subtypes, for those tumours expressing EGFR. We chose HT1080 fibrosarcoma for our *in vivo* study because it is one of the common representative cell lines in STS studies in the literature, it expresses the target and is the most sensitive cell line of all in terms of lower IC_50_s for both drugs in our *in vitro* study (Table 1).

In 2000, the injectable form of arsenic trioxide (trisenox) was approved by the US Food and Drug Administration for the treatment of patients with acute promyelocytic leukemia (APL) [62]. Arsenic trioxide causes tumour cell differentiation, triggers cell apoptosis and induces G1 and/or G2-M phase arrest via down-regulation of CDK2, CDK6, cyclin D1, Cyclin E, Cyclin A and Cdc2 kinase [48]. Considering that organo-arsenicals are often more stable, less toxic and excreted more rapidly than inorganic arsenical, we produced a tripeptide trivalent arsenical, 4-(N-(S-glutathionylacetyl) amion) phenylarsenoxide (GSAO). GSAO is cleaved by γ-glutamyl transpeptidase at the cell surface to produce 4-(N-(S-cysteinylglycylacetyl) amino) phenylarsonous acid (GCAO) [63]. GCAO enters the cell via an organic ion transporter and is further processed by dipeptidases to 4-(N-(S-cysteinylacetyl) amino) phenylarsonous acid (CAO) in the cytosol. Exporting CAO from cells is controlled by the multidrug-resistance associated protein isoforms 1 and/or 2 (MRP1/2) [64]. A first-in-human phase I clinical trial demonstrated that GSAO was cleared extremely quickly from plasma (mean half-life 10.1 minutes) with no evidence of accumulation [65]. These studies prompted the development of a second-generation organo-arsenical compound PENAO, which accumulates in cells 85-fold faster than GSAO because of increased rate of entry (due to no requirement for processing by γ-glutamyl transpeptidase at the cell surface) and less efficient of export by MRP1/2 [45]. PENAO, similarly as CAO, enters the mitochondrial matrix and inactivates ANT in the mitochondrial inner membrane and triggers Ca^2+^-dependent MPTP, by cross-linking the matrix facing thiols of Cys^57^ and Cys^257^ in ANT [39, 44]. Inactivation of ANT causes proliferation arrest, ATP depletion, superoxide level increase, mitochondrial depolarization and apoptosis in proliferating endothelial and tumour cells. Similar to our study in diverse STS and OS cell lines, PENAO showed anti-proliferation with low micromolar IC_50_s in epithelial cancer cells [45, 51]. A Phase I/IIa dose escalation study of PENAO in patients with solid tumours refractory to standard therapy is currently recruiting.

Previous studies have shown sensitivity of gynaecological, breast and lung cancer cell lines to gefitinib monotherapy in a clinically relevant setting, with IC_50_ values ranging from 1 – 10 µM [60, 66, 67]. However, when used on a panel of 12 sarcoma cell lines, we found that gefitinib alone was not sufficient to arrest proliferation (IC_50_ > 14 µM). We also demonstrated that PENAO alone had similar anti-proliferative activity in the panel of 12 sarcoma cell lines (IC_50_: 1.1–7.3 µM) compared to those previously reported for arsenic trioxide on sarcoma cells [58, 68]. Recently, four pre-clinical studies showed interesting combination profiles of EGFR inhibitors and inorganic arsenical-based drugs (sodium arsenite and arsenic trioxide) on melanomas [55], acute promyelocytic leukemia [56], hepatocellular carcinoma [57] and diverse solid cancer types including two osteosarcoma cell lines [58]. Therefore, we investigated whether gefitinib and our organic arsenical-based metabolism inhibitor PENAO had synergistic effect on cell proliferation, apoptosis and metabolism in a panel of twelve sarcoma cell lines and in an orthotopic fibrosarcoma xenograft mouse model.

Combined treatments were performed with the aim of eventually reaching anti-tumour efficacy at lower doses of each single agent. In the fixed ratio setting of the combination therapy, both types of proliferation assays (real-time and end-point MTT) provided consistent results demonstrating synergism between the drugs. A 2–10 fold dose reduction index (DRI) of PENAO and 1.5–2 fold of gefitinib was achieved after combination therapy, demonstrating that PENAO and gefitinib combination treatment had a synergistic inhibitory effect capable of overcoming the insensitivity of chosen sarcoma cell lines. The DRI obtained for PENAO used efficacious doses (0.1–1.8 µM) in the range of what would be achievable in animal tumour model settings (Dilda et al., unpublished data).

We initially examined *in vitro* growth inhibition and the benefit of the combination in a panel of sarcoma cell lines. Once this was demonstrated, we focused upon a representative cell line for the complex and costly mechanism studies. We chose concentrations of the single agents to have no or limited effects on cell death. By contrast PENAO and Gefitinib combination treatment significantly induced early and late apoptosis on HOS cells when compared to the control and single drug treatment groups. Previous studies on leukaemia cell lines demonstrated that arsenic trioxide single treatment can induce apoptosis and decrease AKT activity via caspase-mediated degradation [69]. Furthermore, arsenite in combination with the PI3K/AKT inhibitor, LY294002, dramatically accelerated arsenite-induced apoptosis in different melanomas [70], justifying further combination therapy that targets the PI3K/AKT pathway. Our group has reported that, in seven STS cell lines, EGFR-targeted monotherapy strikingly inhibited the activities of EGFR and downstream PI3K/AKT, despite showing limited anti-proliferation [26]. Two studies [55, 58] showed that EGFR inhibitors AG1478 and erlortinib significantly upregulated apoptosis induced by inorganic AsBD arsenite and arsenic trioxide on various cancer cells, via a suppression of EGFR phosphorylation and a substantial inhibition of phosphorylated-AKT levels [55]. We have now demonstrated a similar effect in sarcoma cell lines with PENAO. The mechanisms via which apoptotic pathways are activated after combination treatment are complex. Several prominent apoptotic actions of EGFR/PI3K/AKT and arsenite may be involved such as caspase-8, -9, nuclear factor kappa B (NF-κB) and Heme oxygenase-1 (HO-1) [55]. A previous study of EGFR inhibitors and sodium arsenite combination treatment reportedly induced both apoptotic and necrotic events in melanoma cells [55].

Cellular metabolism is an important component of cancer growth [37], with tumour cells demanding a higher rate of glucose carbon required for energy [34]. Cancer cells have the ability to shift their metabolism from oxidative phosphorylation to glycolysis to generate ATP, resulting in increased acid production [33]. This encouraged us to explore PENAO in this combination. We demonstrated that HOS cells displayed a reasonable inhibition in mitochondrial activity (decreased OCR) after PENAO single agent treatment, which was similarly observed in glioblastoma and diffuse intrinsic pontine glioma [50, 52, 71, 72]. Our results also showed a significant reduction of OCR and increase of ECAR in HOS cells treated with PENAO plus gefitinib when compared to single agents. These changes in mitochondrial respiration were observed after 24 hours of treatment. It is postulated that mitochondrial perturbations resulting in increased ECAR are early events in the process of proliferation arrest and cell death [73].

In conclusion, concurrent combination treatment of PENAO and gefitinib on sarcoma cells synergistically decreased cell proliferation, significantly induced apoptosis and altered mitochondrial function *in vitro* and in an orthotopic human fibrosarcoma xenograft mouse model. With PENAO presently in clinical phase I, this outcome will encourage the design of subsequent phase II trials to improve the limited treatment options for sarcoma patients.

## MATERIALS AND METHODS

### Drugs & corresponding solvents

Gefitinib (IRESSA, ZD1839) powder (AstraZeneca, London, UK) was dissolved in dimethyl sulfoxide (DMSO, Sigma-Aldrich, Castle hill, NSW, Australia) at a stock concentration of 50 mM, kept at –20°C and thawed when required. PENAO powder, synthesised by Dr. Reddy’s Laboratories (Hyderabad, India), was dissolved in buffer (0.14 M NaCl, 20 mM HEPES, 20 mM Glycine, 1 mM EDTA in milliQ H_2_O, pH 7.0) at a stock concentration of 12–15 mM. In aqueous solution, the trivalent arsenic moiety of PENAO can oxidise into an inactive form (pentavalent arsenical moiety), thus was kept at 4°C and used for 14 days before being remade. PENAO reactivity was tested weekly via arsenical titration against 2,3-dimercaptopropanol (DMP), a synthetic di-thiol. Calculations were based on unbound thiols reacting with 5,5′-dithiobis (2-nitrobenzoic acid) (DTNB). The absorbance of the DMP/DTNB derivative was then read at 412 nm with a spectrophotometer (SpectraMax 190, Molecular Devices, CA, USA).

### Cell culture

The GCT, HT1080, SW872, SW982, Saos-2 and MG63 were purchased from the American Type Culture Collection (VA, USA). The 778 and 449B were kindly supplied by Florence Pedatour from Nice University Hospital (Nice, France). The 143B, U2-OS, HOS and SJSA were generously provided by David Thomas from Peter MacCallum Cancer Centre (Melbourne, Australia). Cells were incubated at 37°C with 95% air atmosphere and 5% CO_2_ in Roswell Park Memorial Institute (RPMI)-1640 media supplemented with 2.0 mM L-glutamine, 1% v/v penicillin/streptomycin and 10% v/v fetal bovine serum.

### Cell proliferation assays

xCELLigence real-time proliferation assay: Cells were seeded onto an E-plate (Roche, Mannheim, Germany) and given 24 hours to adhere to the bottom of the well before being treated with vehicle, gefitinib or PENAO as single agents and in combination at appropriate dilutions. The vehicle control for gefitinib was DMSO at the highest concentration used for gefitinib treatment, control for PENAO was the buffer (0.14 M NaCl, 20 mM HEPES, 20 mM Glycine, 1 mM EDTA in milliQ H_2_O, pH 7.0), and for the combination, both DMSO and buffer were used as the experimental control. E-Plate was placed on the xCELLigence real-time cell analyser multiple-plate (RTCA MP) instrument (Roche, Mannheim, Germany) in an incubator at 37°C with 95% air atmosphere and 5% CO2. Cell index (impedance proportional to the number of viable cells) was measured every 5 hours for 95 hours.

MTT end-point proliferation assay: MTT (3-(4,5-dimethylthiazolyl-2)-2,5-diphenyltetrazolium bromide) salts were dissolved in phosphate-buffered saline (PBS) at 5 mg/ml and then filtered to remove insoluble residue. Cells were seeded and given 24 hours to adhere to a standard 96-well plate and then treated with the appropriate drug dilutions. When combined, the drugs were in a constant ratio with eight different dose combinations. After 48–72 hours of contact with treatment, cells were incubated with MTT at 37°C for 4 hours. Mitochondria of viable cells cleave the tetrozolium ring [59] to form insoluble MTT formazan crystals. Without disturbing the cells, media and MTT was carefully removed. DMSO was added to dissolve the crystals and contents were homogenised with a plate shaker for 10 minutes at room temperature. Absorbance was read with the spectrophotometer (Molecular Devices, CA, USA) at 550 nm. All treatments were normalized to the control (untreated cells) and expressed as a percentage of the control.

Combination index (CI) and dose reduction index (DRI) values were calculated by the Chou-Talalay equation [74] using CalcuSyn software (Biosoft, Cambridge, UK). CI < 1 indicates synergism. DRI is the measure of how much the dose of each treatment in a synergistic combination can be reduced compared with the doses of each treatment alone.

### Annexin V/PI flow cytometry assay

Cells were seeded in 6-well plates (100,000 cells/well), given 24 hours to reattach and treated with the appropriate drug dilution and combination for 24 hours. All cells from the plate (detached with trypsin/EDTA) were extracted and centrifuged at 200 × g for 5 minutes. The Annexin-V-FLUOS Staining Kit (Roche, Mannheim, Germany) was used to detect early and late apoptotic cells. The pelleted cells were resuspended in 100 µl of AnnV/PI solution, incubated in the dark for 15–25 minutes at room temperature, and then diluted with 500 µl of HEPES buffer and ready to be analysed on the FACSCanto II flow cytometer (Becton Dickinson, NJ, USA).

### Real-time metabolic analysis assay

HOS cells were seeded onto a XF^e^24 plate and placed in an incubator at 37°C with 95% air atmosphere and 5% CO_2_. After 24 hours, cells were treated with the appropriate drug dilution and combination. Sensor cartridge was soaked in calibration buffer overnight in an incubator without CO_2_ at 37°C. 24 hours after treatment, media (RPMI-1640) was replaced with DMEM XF assay media without pH buffering capacity. XF^e^24 plate was placed in an incubator without CO_2_ at 37°C for 1 hour. Calibrated sensor and XF^e^24 plate were placed in the Seahorse XF^e^ Extracellular Flux Analyzer (BioScience, Hohenkammer, Germany) to measure the oxygen consumption rate (OCR) and extracellular acidification rate (ECAR) in each well.

### Animal experiments

All animal experiments were approved by UNSW Animal Care and Ethics Committee. Ten-week Balb/c nude mice were obtained from the Animal Resources Centre (Perth, Australia). Based on our previous optimisation test [26], 0.1 × 10^6^ HT1080/mouse were intramuscularly injected into the right back leg. After 24 hours, mice were randomly divided into four groups (*n* = 9–10 per group) and treated by gefitinib (20 mg/kg via gavage daily), PENAO (3 mg/kg/day via micro-osmotic pump for continuous systemic administration, Alzet model 2002, Cupertino, CA), combination or vehicle control (DMSO via gavage daily and PENAO buffer via micro-osmotic pump continuously). The pumps were implanted subcutaneously in the flank and delivered vehicle or 3 mg/kg/day PENAO. Mice were monitored daily for any loss of condition, and tumour progression was documented by measurements using electronic callipers in two dimensions (d1 and d2) and the volume (V) was calculated by the standard formula for an ellipse: V = 1/6 π(d1*d2)^3/2^. There were two different end-points: 1) all mice were sacrificed on 20-day post-treatment, and 2) individual mouse was sacrificed once its tumour reached about 1000 mm^3^ (*n* = 6 per group), according to ethics considerations. Tumour growth inhibition (TGI) was calculated by the formula: TGI (%) = (V_c_-V_t_)/V_c_*100, where V_c_, V_t_ are the average tumour volume of control and treated groups at the end of the study. Tumour volume quadrupling time (TVQT) were estimated using interpolation from the best fit from a nonlinear regression curve fitting an exponential growth curve. Growth delay was calculated as C-V where C and V were times in days for mean tumour size in the combination (C) and vehicle (V) groups to reach 400% the initial tumour volume [75]. At the end points, blood was collected, and serum was sent to South Eastern Area Laboratory Services for toxicity test by examining the liver function (Alanine Amino Transferase, ALT and Alkaline Phosphatase, ALP) and kidney function (urea, creatinine).

### Statistical analysis

Values are presented as mean ± standard deviation (single population) or mean ± standard error of the mean (SEM) (multiple groups). Multiple categorical data was analysed by non-parametric Kruskal-Wallis and post-hoc Tamhane’s test. Multiple quantitative data was analysed by one-way ANOVA (analysis of variance), or a general linear model of multivariate analysis of variance (MANOVA) and post-hoc Bonferroni test (data assumed to be normally distributed) where *p* (2-tail) < 0.05 was considered statistically significant. Time-dependent survival data were analysed by Cox progression and log rank test. Statistical analyses were performed using IBM SPSS Statistics 24 software (IL, USA).
